# LC3-Associated Phagocytosis Is Required for Dendritic Cell Inflammatory Cytokine Response to Gut Commensal Yeast *Saccharomyces cerevisiae*

**DOI:** 10.3389/fimmu.2017.01397

**Published:** 2017-10-25

**Authors:** Dimitra Lamprinaki, Gemma Beasy, Aleksandra Zhekova, Alexandra Wittmann, Steve James, Jo Dicks, Yoichiro Iwakura, Shinobu Saijo, Xiaomin Wang, Chung-Wai Chow, Ian Roberts, Tamas Korcsmaros, Ulrike Mayer, Thomas Wileman, Norihito Kawasaki

**Affiliations:** ^1^Food and Health Institute Strategic Programme, Quadram Institute, Norwich, United Kingdom; ^2^Norwich Medical School, University of East Anglia, Norwich, United Kingdom; ^3^The National Collection of Yeast Cultures, Quadram Institute, Norwich, United Kingdom; ^4^Centre for Animal Disease Models, Research Institute for Biomedical Sciences, Tokyo University of Science, Chiba, Japan; ^5^Department of Molecular Immunology, Medical Mycology Research Center, Chiba University, Chiba, Japan; ^6^University of Toronto, University Health Network, Toronto, ON, Canada; ^7^Earlham Institute, Norwich, United Kingdom; ^8^Gut Health and Food Safety Programme, Quadram Institute, Norwich, United Kingdom; ^9^School of Biological Science, University of East Anglia, Norwich, United Kingdom

**Keywords:** LC3-associated phagocytosis, dendritic cell-associated lectin 2, fungi, dendritic cell, autophagy

## Abstract

The human fungal microbiota known as mycobiota is increasingly recognized as a critical factor in human gut health and disease. Non-pathogenic commensal yeasts such as *Saccharomyces cerevisiae* promote homeostasis in the gut, whereas dysbiosis of the gut mycobiota is associated with inflammation. Glycan-binding receptors (lectins) are key host factors in host–mycobiota interaction in the gut. They are expressed on immune cells such as dendritic cells (DCs) and recognize fungal polysaccharides. This interaction is imperative to mount appropriate immune responses for immune homeostasis in the gut as well as clearance of fungal pathogens. Recent studies demonstrate that microtubule-associated protein light-chain 3 (LC3)-associated phagocytosis (LAP) is involved in lectin–fungi interactions. Yet, the biological impact of LAP on the lectin function remains largely elusive. In this report, we demonstrate that in mouse LAP is linked to dendritic cell-associated lectin 2 (Dectin-2), a C-type lectin specific to fungal α-mannan polysaccharide. We found that mouse Dectin-2 recognizes commensal yeast *S. cerevisiae* and *Kazachstania unispora*. Mouse bone marrow-derived DCs (BMDCs) produced inflammatory cytokines TNFα and IL-1β in response to the yeasts in a Dectin-2 and spleen tyrosine kinase (Syk)-dependent manner. We found that *S. cerevisiae* and *K. unispora* induced LAP in mouse BMDCs upon internalization. Furthermore, LC3 was activated by stimulation of BMDCs with the yeasts in a Dectin-2 and Syk-dependent manner. To address the biological impact of LAP on Dectin-2 yeast interaction, we established a knock-in mouse strain (Atg16L1^E230^, thereafter called E230), which BMDCs exhibit autophagy-active and LAP-negative phenotypes. When stimulated with yeasts, E230 BMDCs produced significantly less amounts of TNFα and IL-1β. Taken together, we revealed a novel link between Dectin-2 and LAP that enables host immune cells to respond to mycobiota.

## Introduction

The human gut contains various fungal species, which form together the fungal microbiota or mycobiota. The number of intestinal commensal fungi represents relatively small fraction of human microbiota when compared to bacteria; however, they play critical roles in gut health and disease (1). Mycobiota dysbiosis is associated with exacerbated gut inflammation in mouse and humans (2, 3). The most dominant fungal species are *Candida albicans* and *Candida tropicalis* in humans and mice, respectively (1, 4, 5). Both species affect the severity of inflammatory bowel diseases. In several human randomized clinical trials, once taken orally, a number of commercially available strains of *Saccharomyces cerevisiae* known as *S. boulardii* has been shown to prevent *Clostridium difficile*-associated diarrhea (6). However, molecular and cellular mechanisms underpinning the impact of mycobiota remain largely unknown.

One of the key host factors includes cell surface lectin receptors expressed on immune cells. Such receptors recognize fungal polysaccharides and induce immune cell activation. Fungal β-glucan is recognized by dendritic cell-associated lectin-1 (Dectin-1) (7), chitin by macrophage mannose receptor (MR) (8), β-mannan by Galectin-3 (9), and α-mannan by dendritic cell-associated lectin 2 (Dectin-2) (10). Dectin-2 possess exclusive specificity to mannose, as all the identified glycan ligands for Dectin-2 contain mannose (10–12). Dectin-2 binding to fungi activates the spleen tyrosine kinase (Syk) signaling pathway and induces immunomodulatory function of DCs including downstream cytokine production (13). This, in turn, induces a Th17 response to remove invasive fungi such as *C. albicans* (14, 15). Another key function of Dectin-2 is internalization of the ligand into the cell. An earlier study shows that anti-Dectin-2 antibody (Ab) binding induces rapid receptor internalization (16), and recently it is shown that Dectin-2 directs intracellular cargo to lysosomes (15). While these reports demonstrate that Dectin-2 is an endocytic/phagocytic receptor, how this pathway impacts on the immunomodulatory function of Dectin-2 remains unknown.

Another key host factor involved in host–fungi interactions is microtubule-associated protein light-chain 3 (LC3)-associated phagocytosis (LAP). LC3 is originally characterized as an autophagy-associated protein that initiates the formation of the autophagosome (17). Cellular stresses such as nutritional change, infection, and oxidative damage recruit LC3 to newly synthesized membrane components that encapsulate damaged organelles and intracellular pathogens for degradation (17). Recent reports indicate that LC3 is also involved in phagocytosis, to fuse phagosomes with lysosomes, thereby facilitating degradation of exogenous components such as invasive fungi. In an *Aspergillus fumigatus* infection model in mouse, LAP is shown to be critical for fungal killing (18). LAP-deficient and autophagy-active bone marrow-derived macrophages (BMMs) exhibit reduced fungal killing (18). LC3β KO bone marrow-derived DCs (BMDCs), which lack both autophagy and LAP, were less efficient in presenting fungal antigens to MHC class II pathway (19). Furthermore, LC3β KO BMMs show enhanced inflammatory cytokine production in response to *C. albicans* (20). Since LC3β KO cells are both autophagy and LAP-negative, these findings need to be confirmed in an autophagy-active and LAP-negative system to assess the role of LAP in immune cell response to fungi.

In this report, we employed a new genetic tool to assess the link between LAP and Dectin-2 in DC–mycobiota interaction. We found that mouse Dectin-2 recognizes two human commensal yeasts *S. cerevisiae* and *Kazachstania unispora*. These yeasts induced the cytokines TNFα and IL-1β from BMDCs in a Dectin-2 and Syk-dependent manner. Using a transgenic mouse strain with a mutation in a key autophagy gene, *Atg16L1*, which is autophagy-active and LAP-deficient (Atg16L1^E230^, thereafter called E230), we found that LAP was required for the inflammatory cytokine production from BMDCs in response to the commensal yeasts. The commensal yeasts induced LAP, shown by the recruitment of LC3 to the internalized yeasts in WT BMDCs, but not in E230 BMDCs. Consistently, we found these yeasts induced LC3 activation and LAP in a Dectin-2-dependent manner. Taken together, our data demonstrate for the first time that interaction between Dectin-2 and commensal non-pathogenic yeast induces LAP, which is required for immunomodulatory function of Dectin-2 in DCs.

## Materials and Methods

### Mice

C57BL/6J WT, Atg16L1^E226^, and Atg16L1^E230^ mice (thereafter called E226 and E230 mice, respectively), which are defective in autophagy and/or LAP, respectively, and Dectin-2 KO mice were maintained in the specific pathogen free animal facility at the University of East Anglia (Norwich, UK). Generation and characterization of E226 and E230 mice will be described elsewhere. The inducible Syk^flox/flox^/rosa26CreERT2 mouse strain was maintained at Toronto University (Toronto, ON, Canada) (21). The tamoxifen induction protocol and characterization of the Syk knockout phenotype have been described (22, 23). Control littermates were treated with 10% v/v ethanol in sunflower oil, the diluent for tamoxifen. Bone marrow cells were harvested and frozen. Animal use in this study was conducted under the project license (70/8177 and 70/8332) authorized by the UK home office, and University of Toronto Faculty Advisory Committee on Animal Services and Toronto General Research Institute Advisory Committee on Animal Services, and conducted in accordance with the guidelines of the Canadian Council on Animal Care.

### Reagents

Chemicals were obtained from Sigma-Aldrich (St. Louis, MO, USA), unless otherwise stated. *N*-succinimidyl ester conjugated (NHS)-Alexa647 and Cell trace violet (CTV) were from ThermoFisher Scientific (Waltham, MA, USA). α-Mannan from *Malassezia furfur* was purchased from InvivoGen (San Diego, CA, USA). Scleroglucan (β-glucan) was obtained from Elicityl (France). Lipopolysaccharide (LPS) from *Klebsiella pneumoniae* O1 was obtained from Dr. Chris Whitfield (University of Guelph, Canada). Alexa647-labeled anti-mouse Dectin-1 Ab (clone 2A11, rat IgG2b) was purchased from Bio-Rad (Hercules, CA, USA). Alexa647-labeled anti-Dectin-2 Ab (clone 2B4, rat IgG2a) was generated as described previously (11). Biotinylated anti-mouse Dectin-2 Ab (clone 2B4) was also generated using NHS-LC-biotin (ThermoFisher Scientific). Alexa647-labeled isotype-control Abs, mouse Fc receptor blocking Ab (clone 93, BioLegend), and *R*-phycoerythrin (PE)-labeled streptavidin were purchased from BioLegend (San Diego, CA, USA). ELISA kits for mouse TNFα and IL-1β were from BioLegend and R&D systems (Minneapolis, MN, USA), respectively. Anti-LC3 A/B (#4108S), β-actin (#4970S), and anti-rabbit IgG conjugated with horseradish peroxidase (HRP) (#7074P2) were from Cell Signaling Technologies (Danvers, MA, USA).

### Cell Lines

RAW macrophage cell line expressing mouse Dectin-2 was given from Dr. Kiyoshi Ariizumi (UT Southwestern, Dallas, TX, USA) and was cultured in RPMI1640 (Lonza, Walkersville, MD, USA) supplemented with 25 mM HEPES, 2 mM l-glutamine, 100 U/mL penicillin/streptomycin, 10% FBS (ThermoFisher Scientific), and 55 µM mercaptoethanol (R10). BWZ.36 cells expressing wild-type mouse Dectin-2 (Dectin-2^WT^), carbohydrate-binding incompetent mouse Dectin-2 (Dectin-2^QPD^), and the mock transfectant were maintained as described previously (11).

### Yeasts

*Saccharomyces cerevisiae* isolated from human feces (#2966) and *C. albicans* (#3779) was from National Collection of Yeast Culture (Norwich, UK). *K. unispora* isolated from human feces (#CBS 3004) was obtained from CBS-KNAW Fungal Biodiversity Centre (Netherlands). Yeasts were initially cultured in yeast media medium at 25°C for 3 days. On the day of experiment, fungi culture was diluted at 1:20 and grown at 37 and 30°C for *S. cerevisiae* and *K. unispora*, respectively. Yeast culture was in exponential growth phase (OD = 0.7) when harvested for subsequent experiments.

### Reporter Assay

Reporter assay was performed as previously described (11). Briefly, 1 × 10^5^ of BWZ.36 cells expressing mouse Dectin-2 were incubated with living yeasts at the indicated multiplicity of infection (MOI). Cells were lysed, and β-galactosidase activity was monitored by a colorimetric assay (11).

### Flow Cytometry

For the binding assay of yeasts to Dectin-2-RAW cells, yeasts were suspended in PBS at 1 × 10^7^ cells/ml and fluorescently labeled with 10 µg/ml NHS-Alexa647 for 1 h at 25°C. Mouse Dectin-2-RAW cells (1 × 10^7^ cells/ml) were labeled with 0.3 µM CTV in PBS for 10 min at 25°C. After labeling, 2.0 × 10^5^ of Dectin-2-RAW cells were incubated with the fluorescent yeasts (MOI = 5) for 1 h at 37°C in FACS Buffer (HBSS containing 25 mM HEPES and 0.1% BSA) supplemented with 2 mM CaCl_2_ or 10 mM EGTA. Cells were washed and analyzed by Fortessa cell analyzer. To analyze lectin expression on mouse BMDCs, cells were incubated with Fc receptor blocking Ab for 10 min at 4°C. The cells were stained with Alexa647-labeled anti-mouse Dectin-1 and Dectin-2 Abs, or the isotype-matched control Abs for 30 min at 4°C. Biotinylated anti-Dectin-2 and the isotype-control Abs were also used in combination with PE-labeled streptavidin. The stained cells were washed with FACS buffer and incubated with 0.33 µg/ml propidium iodide before analysis by Fortessa. All data were processed in FlowJo (TreeStar, USA).

### Analysis of Cytokine Production from BMDCs

Mouse BMDCs used in this study were generated as described before (11). To analyze cytokine production, 1 × 10^5^ of BMDCs were cultured in a 96-well round-bottom plate in the presence of *S. cerevisiae, K. unispora*, and *C. albicans* at a MOI of 5, 100 µg/ml α-mannan, or 100 ng/ml LPS, and 1 mg/ml curdlan (Wako Chemicals, Tokyo, Japan), a β-glucan, at final concentration for 16 h. The amount of TNFα and IL-1β in the culture supernatant was measured by ELISA.

### Western Blot Analysis of LC3 Lipidation

Bone marrow-derived DCs were generated in a 24-well plate (1.9 × 10^5^ cells/well). On day 6, *S. cerevisiae* and *K. unispora* were added to the well (MOI = 5 and 10) and incubated for 60 min at 37°C. After incubation, culture supernatant was removed and 80 µl of lysis buffer (20 mM Tris–HCl, 150 mM NaCl, 1 mM EDTA, 1% Triton-X 100, 2 mM sodium orthovanadate, 10 mM sodium fluoride, and one complete ULTRA tablet, Mini) was added to the well and incubated for 5 min at 4°C. After the cell lysis, cell debris was removed by centrifugation at 21.1 × *g* for 10 min at 4°C. The supernatant was collected, mixed with 4× of laemmli buffer (Bio-Rad) containing 1.43 M β-mercaptoethanol, and heated for 15 min at 75°C. Twenty microliters of cell lysate were loaded onto a 4–15% gradient TGX mini gel (Bio-Rad) and ran for 30 min at 200 V. Proteins were transferred to PVDF membrane (Thermo Scientific, Waltham, MA, USA) for 1 h at 100 V. Membrane was blocked with 5% non-fat milk (Sigma-Aldrich) in PBS containing 0.05% Tween-20 (PBS-T) for 1 h at 25°C. Membrane was washed 4 times for 5 min with PBS-T. The membrane was incubated with primary Abs anti-LC3 A/B (1:1,000), β-actin (1:5,000) in PBS containing 1% BSA for 16 h at 4°C. Membrane was washed in PBS-T as above and incubated with anti-rabbit IgG conjugated with HRP (1:3,000) in 5% non-fat milk in PBS-T for 1 h at 25°C. The membrane was washed in PBS-T as above and then incubated with ECL detection reagent (GE Healthcare Life Sciences, Marlborough, MA, USA). Image was obtained and the band intensity was quantified using Fluorochem E (ProteinSimple, San Jose, CA, USA).

### Fluorescent Microscopy

Mouse BM cells were cultured on coverslips (VWR, Radnor, PA, USA) placed in 24-well plate in the culture medium for BMDC generation (11). To test phenotype of E226 and E230 cells, on day 6 BMDCs were either left in the culture medium, incubated in HBSS for 2 h at 37°C to induce starvation-driven autophagy, or incubated with polystyrene beads (Polysciences, Warrington, PA, USA) for 1 h at 37°C and then Monensin (Sigma-Aldrich) for 1 h at 37°C to induce LAP. The cells were washed with PBS three times and fixed with ice-cold 100% methanol for 10 min at −20°C. The cells were washed and incubated with PBS containing 5% goat serum (Gibco, Waltham, MA, USA), 0.3% Triton-X for 30 min at 25°C. The fixed cells were incubated with anti-LC3 Ab (1:500, Cell Signaling, Danvers, MA, USA) in PBS containing 1% BSA, 0.3% Triton-X for 16 h at 4°C on a rocker (20 rpm). The cells were washed with PBS three times and incubated with Alexa488 goat anti-rabbit IgG (1:1,000, Life Technologies, Carlsbad, CA, USA) in PBS containing 1% BSA, 0.3% Triton-X for 2 h at 25°C. The cells were washed with PBS three times and stained with DAPI (1.0 µg/ml, Thermo Scientific) in PBS for 5 min at 25°C on a rocker (20 rpm). The cells were washed twice with PBS, the coverslip was mounted in fluoromount (eBioscience) and kept at 4°C in the dark until being imaged with Zeiss using a software Axio Vision fluorescence imager. Images were taken using 63× objective setting with an immersion oil Type LDF (Cargill, Wayzata, MN, USA). To monitor yeast-induced LAP in BMDCs, yeasts were washed twice with PBS and incubated with 4% paraformaldehyde (PFA) in PBS for 1 h at 25°C. The PFA-fixed yeasts were washed with PBS and suspended at 1.0 × 10^7^ cells/ml in BMDC culture medium. The yeasts were further incubated with 10 µg/ml NHS-Alexa555 (ThermoFisher Scientific) for 1 h at 25°C in the dark and washed with the medium. On day 6, BMDCs were incubated with Alexa555-labeled yeast for 2 h at 37°C at a MOI of 10. Around 100 cells for WT, E226, and E230, BMDCs were analyzed to count the number of starvation-induced LC3 puncta in the cell. The puncta size was defined by a diameter of 1.67 µm, any LC3-punta smaller or larger were not included.

### Statistical Analysis

One-way ANOVA followed by Tukey’s test were used for statistical analysis on Prism software (GraphPad). *p* < 0.05 was considered as statistically significant.

## Results

### Mouse Dectin-2 Recognizes Commensal Yeasts

**Figure 1:**
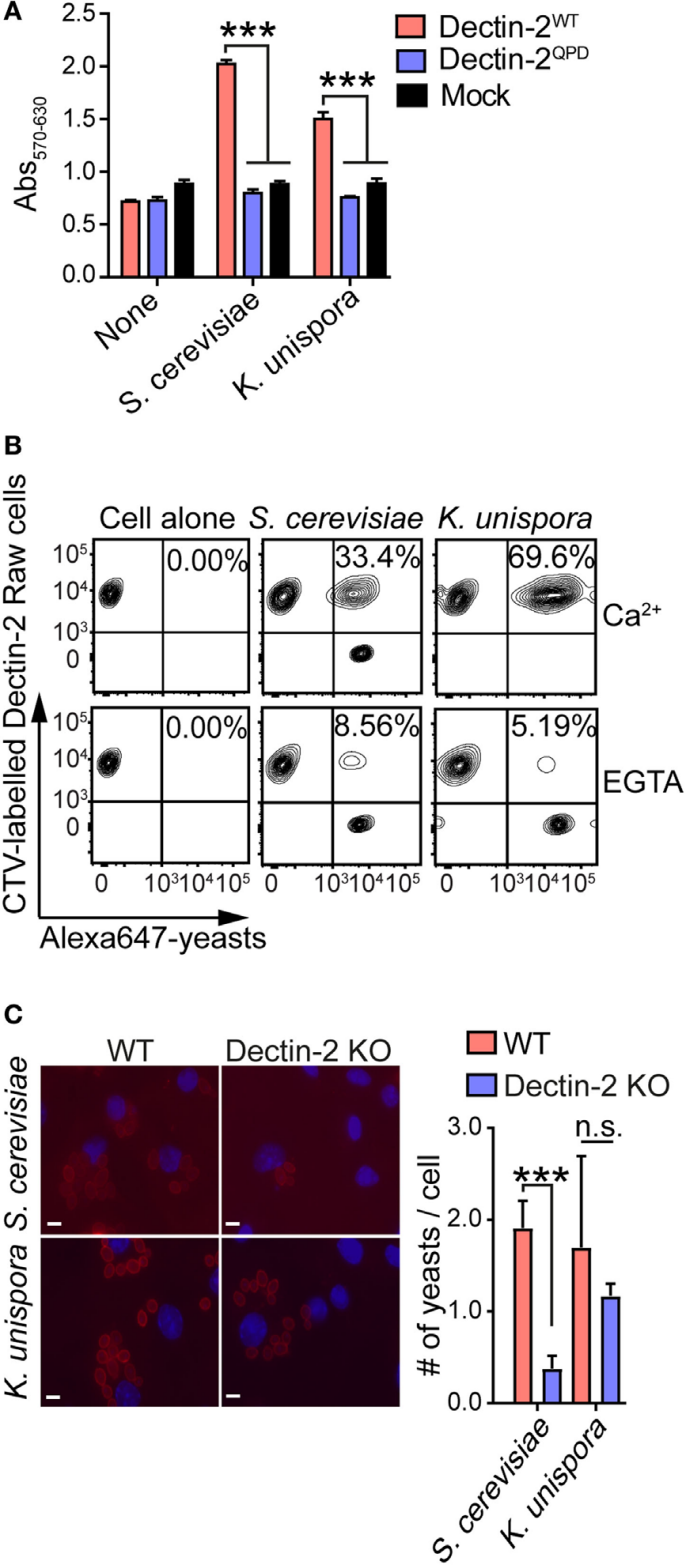
Mouse Dectin-2 binds to gut commensal yeasts. **(A)** BWZ reporter cells expressing mouse Dectin-2^WT^, Dectin-2^QPD^, and mock transfectant were incubated with the indicated yeasts at a MOI of 5. After 1 day incubation, β-galactosidase activity in the reporter cells was monitored by a colorimetric assay. **(B)** Mouse Dectin-2-expressing RAW macrophages were incubated with Alexa647-labeled yeasts or left alone. The binding was analyzed by flow cytometry. **(C)** Mouse BMDCs were incubated with the Alexa555-labeled and paraformaldehyde-fixed yeasts (red) at a MOI of 10. After 2 h incubation, images of BMDCs were taken with the DAPI staining (blue). White bar indicates 5 µm. The number of yeasts associated with BMDCs was quantified. Data shown are the mean of triplicates ± SD from one representative experiment and reproducible in three independent experiments. Statistical analyses were performed by one-way ANOVA followed by Tukey’s test. *p < 0.05; **p < 0.01; ***p < 0.001; n.s., not statistically significant.

We tested Dectin-2 binding to the commensal yeast *S. cerevisiae* and *K. unispora* isolated from human feces. To check the binding of *S. cerevisiae* and *K. unispora* to Dectin-2, we employed the mouse Dectin-2-expressing BWZ.36 reporter cell assay in which we measured β-galactosidase activity as a readout for Dectin-2 interaction with yeasts (11). Importantly, human and mouse Dectin-2 has conserved carbohydrate-binding specificity (10, 24). We found that the commensal yeasts bound to mouse Dectin-2 in the reporter assay (Figure 1A). The binding was mediated *via* the carbohydrate-recognition domain of Dectin-2, as the carbohydrate-binding incompetent mutant of Dectin-2 (Dectin-2^QPD^) failed to bind to the yeasts (Figure 1A) (11). Dectin-2 interaction with the yeasts was further confirmed using RAW macrophage expressing mouse Dectin-2 by flow cytometry. We found that after 1 h incubation with yeasts, Dectin-2-RAW cells bound to both *S. cerevisiae* and *K. unispora* (Figure 1B). Of importance, the binding was diminished when the calcium ion chelator EGTA was added (Figure 1C), indicating Ca^2+^-dependent interaction of Dectin-2 with yeasts. We also tested mouse BMDC interaction with the yeasts. To this end, we incubated mouse BMDCs with Alexa555-labeled yeasts and counted the number of fluorescent yeasts associated with the cell. We found that Dectin-2 KO BMDCs showed fewer number of yeasts associated with the cell when compared to WT BMDCs (Figure 1C). Taken together, these data demonstrate that Dectin-2 recognizes commensal yeasts including *S. cerevisiae* and *K. unispora* through Ca^2+^-dependent glycan recognition.

### Gut Commensal Yeasts Stimulate Mouse BMDCs in Dectin-2 and Syk-Dependent Manner

**Figure 2:**
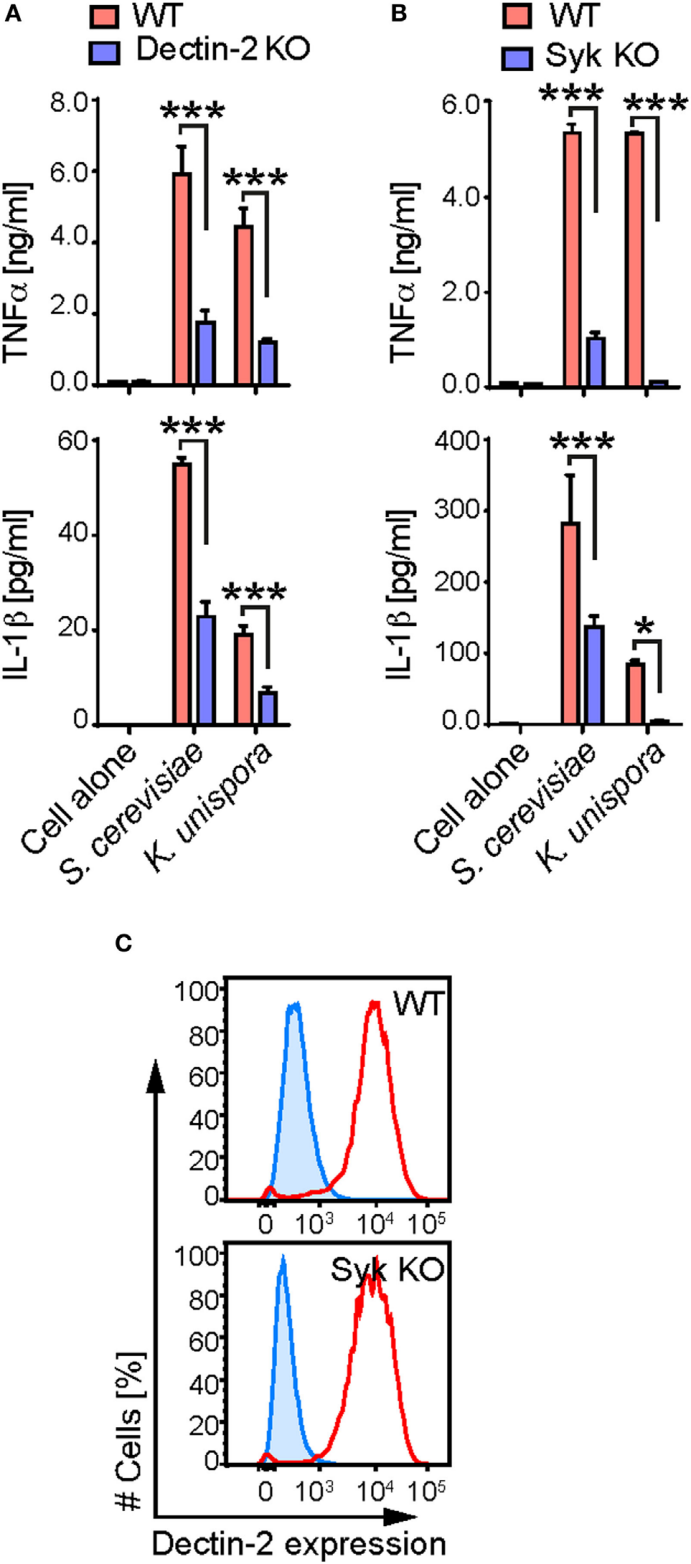
Dectin-2 and Syk-dependent cytokine production by gut commensal yeasts. **(A,B)** WT, Dectin-2, and Syk KO BMDCs were incubated with the indicated yeasts at a multiplicity of infection of 5. After 16 h incubation, the supernatant was harvested, and the amount of TNFα and IL-1β was measured by ELISA. **(C)** WT and Syk KO BMDCs were stained with anti-Dectin-2 Ab (red) or the isotype-control Ab (blue). The stained cells were analyzed by flow cytometry. Data shown are the mean of triplicates ± SD from one representative experiment and reproducible in three independent experiments. Statistical analyses were performed by one-way ANOVA followed by Tukey’s test.

Commensal yeasts and their components are increasingly recognized to stimulate host immune cells, which is important for gut health and disease (2, 4, 5). Therefore, we assessed the contribution of mouse Dectin-2 and its signaling pathway to the immune response to commensal yeasts. We found that *S. cerevisiae* and *K. unispora* stimulated mouse BMDCs, as shown by production of the cytokines, TNFα and IL-1β, after incubation at a MOI of 5 (Figure 2). The response was partially mediated by Dectin-2, as Dectin-2 KO BMDCs showed more than 50% reduction in cytokine production in response to the yeasts (Figure 2A). The residual cytokine production is likely mediated by other fungal recognition receptors including Dectin-1, MR, and TLRs, as shown previously (5, 25). This reduced cytokine production was only seen when Dectin-2 KO cells were stimulated with Dectin-2 ligands, as Dectin-2 KO BMDCs show normal response to ligands for TLR4 and Dectin-1 for instance (Figure S1 in Supplementary Material) (11, 14).

Since Syk is required for intracellular signaling of the C-type lectins including Dectin-2 (13), we sought to assess the contribution of Syk in BMDC response to commensal yeasts. Of note, *K. unispora* failed to stimulate Syk KO BMDCs, suggesting a dominant role of Syk-coupled fungal recognition receptors such as Dectin-1 and Dectin-2 (Figure 2B). Syk KO BMDCs were still able to respond to *S. cerevisiae* but to a lesser extent than WT BMDCs, implying contribution of non-Syk-coupled activation receptors such as TLRs (Figure 2B) (5). We confirmed that Syk KO BMDCs express Dectin-2 at a level indistinguishable with that of WT cells (Figure 2C). Taken together, our data indicate that gut commensal yeasts induce TNFα and IL-1β production from BMDCs in Dectin-2 and Syk-dependent fashion.

### Gut Commensal Yeasts Induce LAP and LC3 Lipidation in BMDCs in a Dectin-2-Dependent Manner

**Figure 3:**
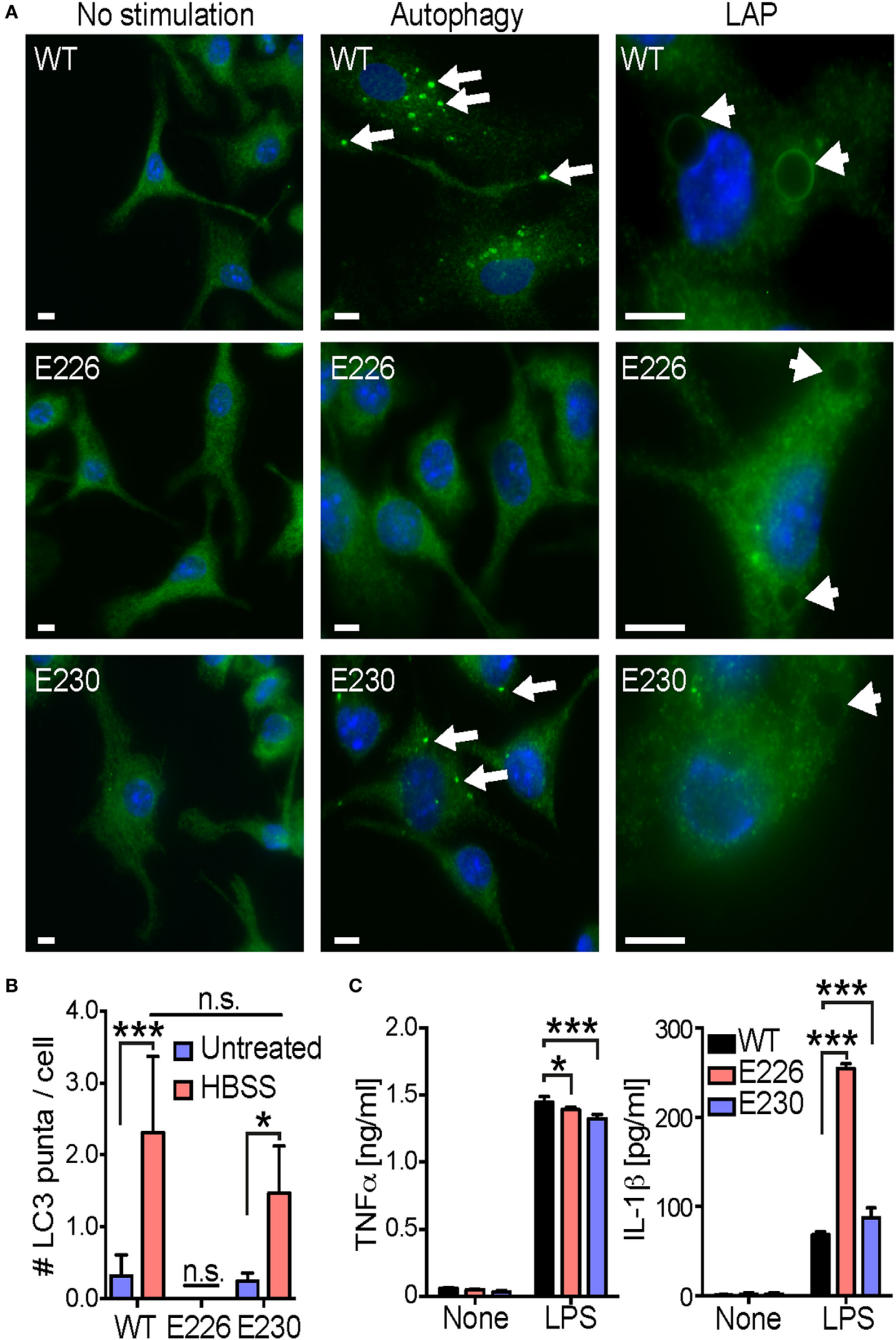
Analysis of autophagy and LAP in E226 and E230 BMDCs. **(A)** The autophagy and LAP phenotype in WT, E226, and E230 BMDCs were analyzed by staining LC3, when cells were starved (autophagy) and polystyrene beads were taken up (LAP), respectively. White bar indicates 5 µm. Arrows and arrow heads show LC3 puncta and internalized beads, respectively. **(B)** The number of starvation-induced LC3 puncta in the cell was counted around 100 cells per each genotype. **(C)** WT, E226, and E230 BMDCs were stimulated with 100 ng/ml of lipopolysaccharide. After 16 h incubation, the supernatant was harvested, and the amount of TNFα and IL-1β was measured by an ELISA. Data shown are the mean of triplicates ± SD from one representative experiment and reproducible in three independent experiments. Statistical analyses were performed by one-way ANOVA followed by Tukey’s test.

Since previous studies suggest that pathogenic fungi *C. albicans* and *A. fumigatus* induce LAP (18, 20), we sought to test whether gut commensal yeasts also do so. To this end, we generated BMDCs from mice carrying mutations in autophagy protein Atg16L1 that affect autophagy and LAP. The E230 mouse strain carries a stop codon at the end of the coiled coil domain that contains glutamate residues E226 and E230 required for autophagy but lacks the linker region and the WD repeat domains required for LAP (26). The E226 mouse strain contains glutamate 226 and two additional amino acids (alanine and glycine) followed by a stop codon, which prevents expression of glutamate at position 230 and is therefore deficient in both autophagy and LAP (26). The autophagy and LAP phenotype of E226 and E230 BMDCs, respectively, were confirmed *in vitro*. E226 BMDCs exhibited the absence of both starvation-induced autophagy and polystyrene bead-induced LAP, whereas E230 BMDCs showed active autophagy to a lesser extent to that of WT and complete absence of LAP (Figures 3A,B). We found that E226 BMDCs produced significantly higher amount of IL-1β in response to LPS (Figure 3C), which was consistent with the previous reports demonstrating the inhibitory role of autophagy in inflammasome activation (17). On the other hand, IL-1β production from E230 BMDCs was almost indistinguishable with that of WT BMDCs, suggesting LAP is not involved in LPS-induced IL-1β production in BMDCs (Figure 3C).

**Figure 4:**
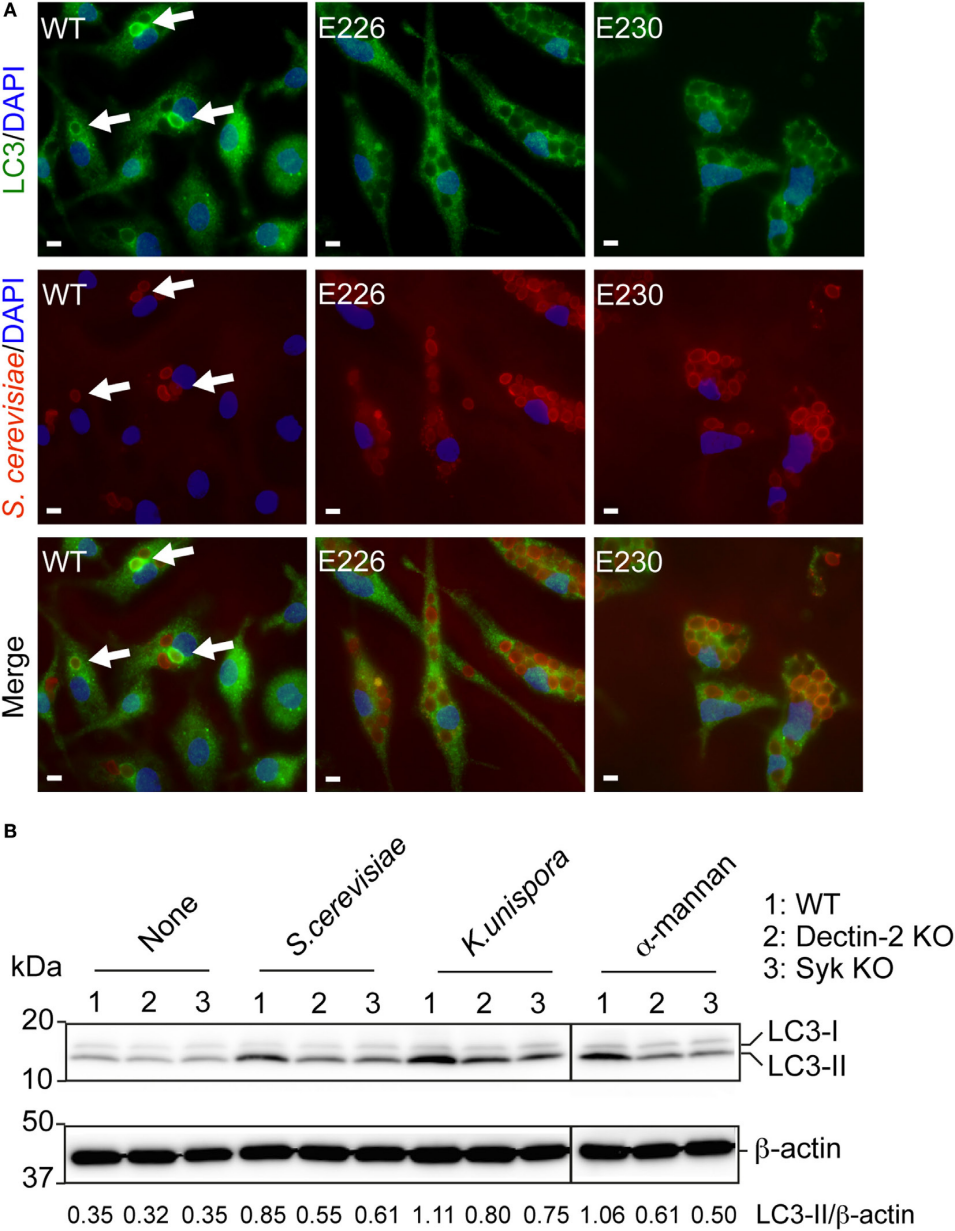
Commensal yeasts induce LAP and LC3 activation. **(A)** WT, E226, and E230 BMDCs were incubated with Alexa555-labeled and PFA fixed *S. cerevisiae* at a MOI of 10 for 2 h. Cells were then fixed, permeabilized, and stained with anti-LC3 Ab and analyzed. White bar indicates 5 µm. Arrows show LC3 recruitment to *S. cerevisiae*. **(B)** WT, Dectin-2 KO, and Syk KO BMDCs were incubated with the indicated yeasts at a MOI of 5 or α-mannan for 1 h. Cells were lysed, and the proteins were separated by SDS-PAGE and blotted to a PVDF membrane. The membrane was probed by anti-LC3 Ab. The band intensity ratio of LC3-II over β-actin is shown in the bottom. Data shown are one representative experiment and reproducible at least two independent experiments.

**Figure 5:**
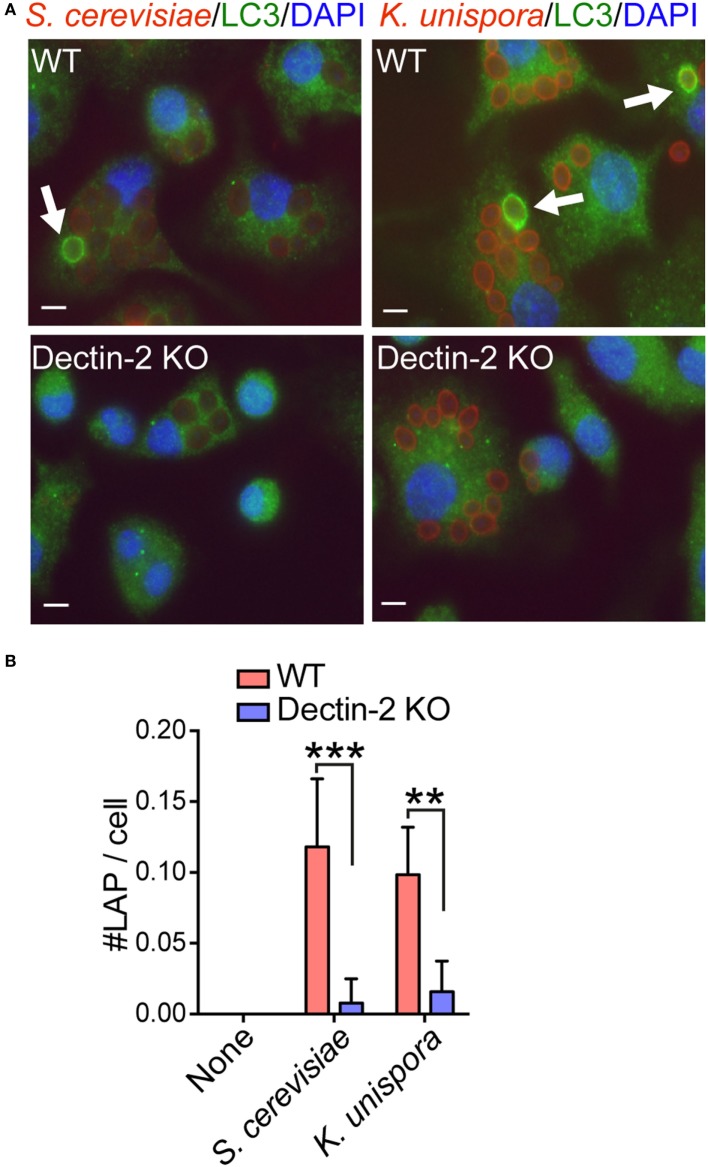
Dectin-2-dependent LAP formation in response to commensal yeasts. **(A)** WT and Dectin-2 KO BMDCs were incubated with Alexa555-labeled and PFA-fixed *S. cerevisiae* and *K. unispora* at MOI 10 for 2 h. White bars indicates 5 μm. Arrows show LC3 recruitment to the yeasts. **(B)** The number of LAP in the cell was counted around 100 cells per each condition. Data shown are one representative experiment and reproducible in three independent experiments. Statistical analyses were performed by one-way ANOVA followed by Tukey’s test.

When WT BMDCs were incubated with PFA-fixed and Alexa555-labeled yeasts, we observed LAP formation in the cell. In WT BMDCs, internalized *S. cerevisiae* was surrounded by LC3, indicating LAP formation (Figure 4A). This was completely abolished in both E226 and E230 BMDCs (Figure 4A). Likewise, Alexa555-labeled and PFA-fixed *K. unispora*-induced LAP in WT BMDCs, but not in E226 nor E230 BMDCs (Figure S2 in Supplementary Material). LC3 is known to be covalently ligated to phosphatidylethanolamine upon autophagy and LAP formation (17). Indeed, we observed lipidation of LC3 in BMDCs upon stimulation with the yeasts, occurring after 1 h incubation (LC3-II in Figure 4B). Of importance, this yeast-induced LC3 lipidation was Dectin-2 dependent, as it was partially reduced in Dectin-2 KO BMDCs (Figure 4B). Furthermore, LC3 lipidation occurred in a Syk-dependent manner (Figure 4B), which was consistent with previous study (20). We also observed LC3 lipidation in response to α-mannan (Figure 4B), suggesting that Dectin-2 ligation is sufficient to induce LC3 lipidation. The α-mannan used in this study bound to Dectin-2, but not Dectin-1 in a reporter assay (Figure S3 in Supplementary Material). Next, we sought to test whether Dectin-2 is involved in LAP formation when cells were stimulated with the commensal yeasts. Indeed, LAP formation in Dectin-2 KO BMDCs was significantly reduced when incubated with *S. cerevisiae* and *K. unispora* (Figures 5A,B). Taken together, these data demonstrate commensal yeasts induce LC3 lipidation and LAP in a Dectin-2-dependent manner.

### LAP Is Required for Inflammatory Cytokine Production from BMDCs in Response to Gut Commensal Yeasts

**Figure 6:**
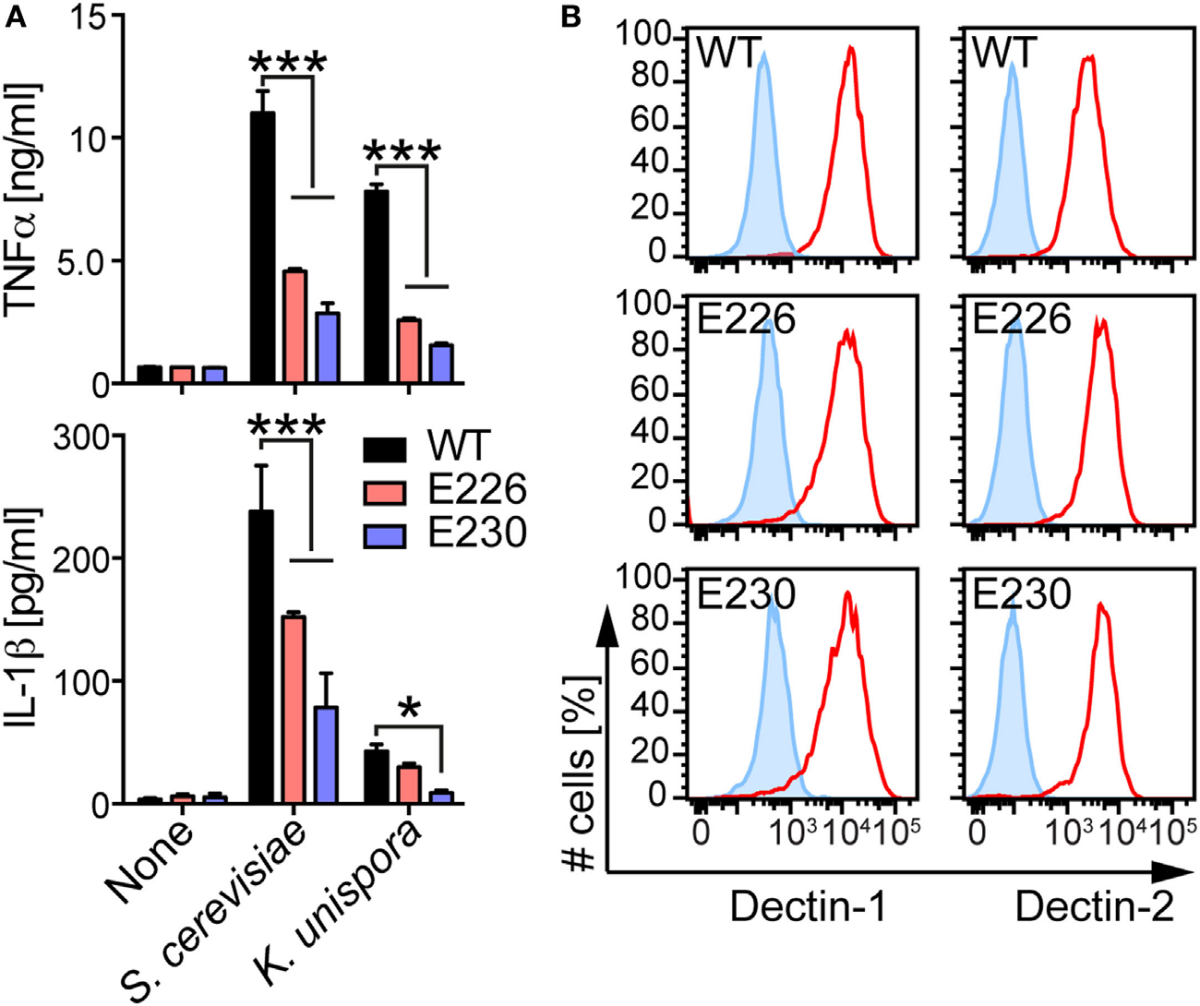
LAP-dependent BMDC cytokine response to commensal yeasts. **(A)** WT, E226, and E230 BMDCs were incubated with the indicated yeasts at a MOI of 5. After 16 h incubation, the supernatant was harvested and the amount of TNFα and IL-1β was measured by ELISA. **(B)** WT, E226, and E230 BMDCs were stained with anti-Dectin-1 and 2 Abs (red) or the isotype control Abs (blue). The stained cells were analyzed by flow cytometry. Data shown are the mean of triplicates ± SD from one representative experiment and reproducible in three independent experiments. Statistical analyses were performed by one-way ANOVA followed by Tukey’s test.

To assess the involvement of LAP in immunomodulatory function of DCs, we compared yeast-induced cytokine production from WT, E226, and E230 BMDCs. When these BMDCs were stimulated with living yeasts, E230 BMDCs produced less amount of TNFα and IL-1β, suggesting that LAP is required for cytokine production in BMDCs in response to the commensal yeasts (Figure 6A). E226 BMDCs also showed modest reduction in both TNFα and IL-1β production in response to *S. cerevisiae*. In case of *K. unispora*-induced cytokine production in E226 BMDCs, reduction in TNFα was statistically significant, but IL-1β not. We observed the reduced TNFα production in E226 and E230 KO BMDCs in response to the PFA-fixed yeasts (Figure S4 in Supplementary Material). While the PFA-fixation of yeasts abrogated the IL-1β response (Figure S4 in Supplementary Material), these data suggest that the reduced cytokine production in E226 and E230 BMDCs was not due to the overgrowth of yeasts. We confirmed that WT, E226, and E230 BMDCs express fungal polysaccharide receptors, Dectin-1 and Dectin-2, at an indistinguishable level (Figure 6B). These data demonstrate that LAP is required for cytokine production in BMDCs in response to commensal yeasts.

## Discussion

The mycobiota is increasingly recognized as a key component of our mucosal surface that affects both bacterial microbiota and host physiology (1). The host immune system is a critical factor that interacts with the mycobiota both directly and indirectly through fungal metabolites and outer membrane vesicles (OMVs) that contain enzymes, nucleic acids, and polysaccharides (1, 27). While recent studies have identified host immune cell receptors responsible for fungal polysaccharide recognition (5), we still do not fully understand the molecular mechanisms underpinning their cell signaling. In this report, we have shown that LAP is involved in Dectin-2 cell signaling induced by gut commensal yeasts.

Commensal non-pathogenic yeasts such as *S. cerevisiae* are habitants in the human gut and provide beneficial effects to both the bacterial microbiota (28) and the host (1). Our results suggest that the commensal yeasts *S. cerevisiae* and *K. unispora* may induce immunomodulatory functions *via* Dectin-2 in the gut. In this regard, our preliminary data indicate that Dectin-2 is expressed in human and mouse myeloid cells in the lamina propria of the small intestine (Wittmann et al., unpublished data). Future studies must include *in vivo* animal models to test whether these commensal yeasts regulate gut immunity upon colonization, and whether Dectin-2 plays any roles in the immune regulation *in vivo*.

The most outer polysaccharide on the fungal cell surface is α-linked mannan, which can be recognized by mannose-specific C-type lectins including MR, DC-SIGN, and Dectin-2 (29). Interestingly, DC-SIGN has been shown to recognize *C. albicans N*-linked α-mannan but not those expressed in *S. cerevisiae* (30), suggesting that fungal surface α-mannan structure can vary among commensal yeasts. On the other hand, Dectin-2 recognizes both *C. albicans* and *S. cerevisiae*, as shown in this study and many others (10, 14). This difference might be explained by the recent structural study on α-mannan recognition by human Dectin-2. Feinberg et al. have suggested that Dectin-2 could recognize internal α-linked mannose residue in α-mannan polysaccharide, while DC-SIGN would only recognize the terminal mannose disaccharide structure because the binding requires 2- and 3-hydroxyl groups of non-reducing terminal mannose, which is not available in the internal mannose residues (24). It is of great interest to determine precise α-mannan structure in *C. albicans* and *S. cerevisiae* to understand molecular determinants that alter host C-type lectin specificity.

Together with previous studies, our data implies that LAP is a common mechanism employed by fungal recognition lectins. Dectin-1-mediated phagocytosis of *C. albicans* or particulated β-glucan induces LAP (19, 20). In this report, we have shown that Dectin-2 is capable of inducing LC3 activation, leading to LAP in response to commensal yeasts (Figure 4). Both Dectin-1 and Dectin-2 induce Syk activation (19) and ROS production (31), which is critical to induce LAP. Based on its association with Syk, we speculate that other fungal recognition lectins such as Mincle and MCL/Dectin-3 are also linked to LAP (32, 33). For this regard, Mincle has been shown to be a phagocytic lectin for *C. albicans* (32). Beside the Syk-coupled receptors, several receptors not linked to Syk also induce LAP. Such receptors include TLRs (34) and TIM-4 which recognizes phosphatidylserine exposed on dead cells (35). TLRs induce ROS production (36), and MyD88 KO macrophages showed impaired ROS production in response to pathogenic bacteria (37). In case of TIM-4, it is not clear whether TIM-4 induce ROS production, or other molecular interactions involved in apoptotic cell recognition do so. Since many other lectins are also phagocytic such as Siglec-1, MR, and DC-SIGN (38, 39), it is of great interest to assess whether they induce ROS production and thereby LAP.

Our results of E226 BMDCs seemed opposite from a previous study showing that LC3β KO BMMs, which lack both autophagy and LAP, produced more TNFα and IL-1β in response to *C. albicans* (20). This might be attributed to the difference in the fungi used. Indeed, consistent with the previous report (20), we found that *C. albicans* induced more IL-1β production in E226 BMDCs compared with that in WT BMDCs (Figure S5 in Supplementary Material). Interestingly, E230 BMDCs showed no reduction in IL-1β response to *C. albicans*, indicating LAP is dispensable for IL-1β response to the fungal pathogen. TNFα response to *C. albicans* was moderately attenuated in E226 and E230 BMDCs (Figure S5 in Supplementary Material). These data indicate that LAP and autophagy pathways may play different roles in response to non-pathogenic yeasts and pathogenic fungi.

Several genetic tools have been utilized to distinguish the precise role of autophagy and LAP pathways. In this regard, autophagy-active and LAP-deficient mice, but not vise versa, exhibit auto-Ab production and kidney dysfunction, similar to the phenotype seen in systemic lupus erythematosus in humans (40), demonstrating a dominant role for LAP over autophagy in the autoimmunity. Furthermore, host–microbiota interaction through microbial OMVs was reported to induce LAP, rather than autophagy (41). Autophagy-active and LAP-deficient DCs failed to induce regulatory T cells in response to OMVs secreted from a commensal bacteria *Bacteroides fragilis* (41). In this report, we present evidence of a LAP-dependent host–mycobiota interaction. Using E230 mice that possess mutation in *Atg16l1* resulting in LAP-deficiency while preserving autophagy, we found that proinflammatory cytokine production of DCs in response to commensal yeasts is dependent on LAP, a response that was not achievable with LC3β KO cells (20). However, we cannot exclude the possibility that ATG16L1 has unknown functions apart from LAP formation, to account for the observed phenotype in E230 BMDCs.

What determines whether cells induce autophagy or LAP? One possible mechanism is the particle size; i.e., smaller size particle induces autophagy, whereas larger size particles are capable of inducing LAP. On the one hand, it is known that TLR ligands such as Pam3 and LPS are capable of inducing autophagy; i.e., LC3 puncta formation in cells (42, 43). On the other hand, when presented with large particles, such as latex beads, these TLR ligands become a potent inducer for LAP (34). Both seem to occur through reactive oxygen species production (34, 42). Consistent with this observation, bacteria (diameter <1 µm) often induce autophagy. Recently, *Pseudomonas aeruginosa* has been shown to induce autophagy rather than LAP (44). Compared to bacteria, fungi are relatively large (diameter >1 µm). Several studies using *C. albicans* and *A. fumigatus* demonstrate that internalization of fungi induces LAP (18, 20). Consistent with these studies, our data demonstrate that commensal yeasts also induce LAP upon interaction with Dectin-2. It is, however, important to mention that autophagy is induced by *C. albicans* and is important for NF-κB activation and eventually fungal killing (45). When we stimulate BMDCs with the fungal polysaccharides, which are smaller than fungi, TNFα production in E226 and E230 BMDCs was indistinguishable with that in WT BMDCs (Figure S5 in Supplementary Material). Enhanced IL-1β production was observed in E226 BMDCs in response to β-glucan, suggesting the inhibitory role of autophagy in β-glucan-induced inflammasome activation. On the other hand, LAP was dispensable for β-glucan-induced IL-1β, as E230 BMDCs exhibited little change (Figure 5 in Supplementary Material). These data also imply that size of the ligands may determine the contribution of autophagy and LAP pathways. For future studies, the polysaccharide probes with defined sizes would be of great help to dissect precise role of autophagy and LAP in host–fungi interactions.

Overall, in this report, we have discovered novel intracellular machinery that enables Dectin-2 to induce cytokine production in DCs. Future study is required to understand the molecular and cellular mechanisms of how LAP and its associated proteins such as ATG16L1 are involved in the Syk-coupled cell signaling pathway.

## Author Contributions

All authors analyzed the data and contributed for preparation of the manuscript. DL, GB, and NK performed experiments, analyzed the data, and prepared the manuscript. AZ provided technical assistance for the work. AW provided technical support in the initiation of this project and established Dectin-1-BWZ cells. SJ, JD, and IR performed yeast analysis and provided technical assistance for yeast culture. YI and SS provided Dectin-2 KO mouse. XW and C-WC provided Syk KO mouse bone marrow. TK and TW provided intellectual support in autophagy pathway. UM supervised generation of E226 and E230 mice. TW and NK supervised and coordinated the work.

## Conflict of Interest Statement

The authors declare that the research was conducted in the absence of any commercial or financial relationships that could be construed as a potential conflict of interest.
