# Supplementary material for: LC3-Associated Phagocytosis Is Required for Dendritic Cell Inflammatory Cytokine Response to Gut Commensal Yeast Saccharomyces cerevisiae
Source: Front Immunol. 2017 Oct 25;8:1397. doi: 10.3389/fimmu.2017.01397 (PMC5661120; doi:10.3389/fimmu.2017.01397)
Supplement: Supplementary file 1 [file Presentation_1.PDF]

Supporting information

LC3-Associated Phagocytosis Is Required for Dendritic Cell Inflammatory Cytokine Response to Gut Commensal Yeast *Saccharomyces Cerevisiae*

Dimitra Lamprinaki[1], Gemma Beasy[1], Aleksandra Zhekova[2], Alexandra Wittmann[1], Steve James[3], Jo Dicks[3], Yoichiro Iwakura[4], Shinobu Saijo[5], Xiaomin Wang[6], Chung-Wai Chow[6], Ian Roberts[3], Tamas Korcsmaros[1,7,8], Ulrike Mayer[9], Thomas Wileman[2,8] and Norihito Kawasaki[1,10]

[1]Food and Health Institute Strategic Programme, Quadram Institute, Norwich, NR4 7UA, UK

[2]Norwich Medical School, University of East Anglia, Norwich UK

[3]The National Collection of Yeast Cultures, Quadram Institute, Norwich UK

[4]Centre for Animal Disease Models, Research Institute for Biomedical Sciences, Tokyo University of Science, Chiba, Japan

[5]Department of Molecular Immunology, Medical Mycology Research Center, Chiba University, Chiba, Japan

[6]University of Toronto, University Health Network Toronto, ON, Canada

[7]Earlham Institute, Norwich, NR4 7UZ, UK

[8]Gut Health and Food Safety Programme, Quadram Institute, Norwich, NR4 7UA, UK

[9]School of Biological Science, University of East Anglia, Norwich, UK

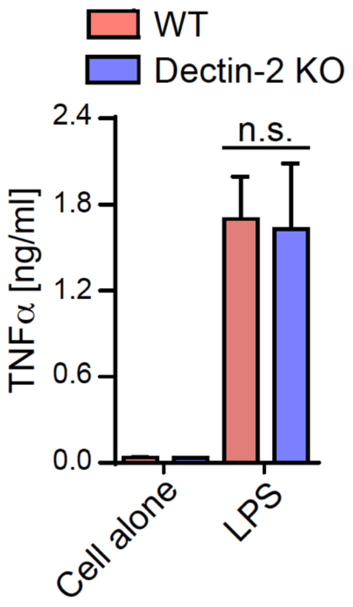

Supplementary Figure 1 Dectin-2 KO BMDCs show normal TNFα production in response to LPS.

WT and Dectin-2 KO BMDCs were stimulated with 4 μg/ml of *K. pneumoniae* O1 LPS. TNFα in the culture supernatant was measured by ELISA. Data shown are the mean of triplicates ± SD from one representative experiment and reproducible at least two independent experiments.

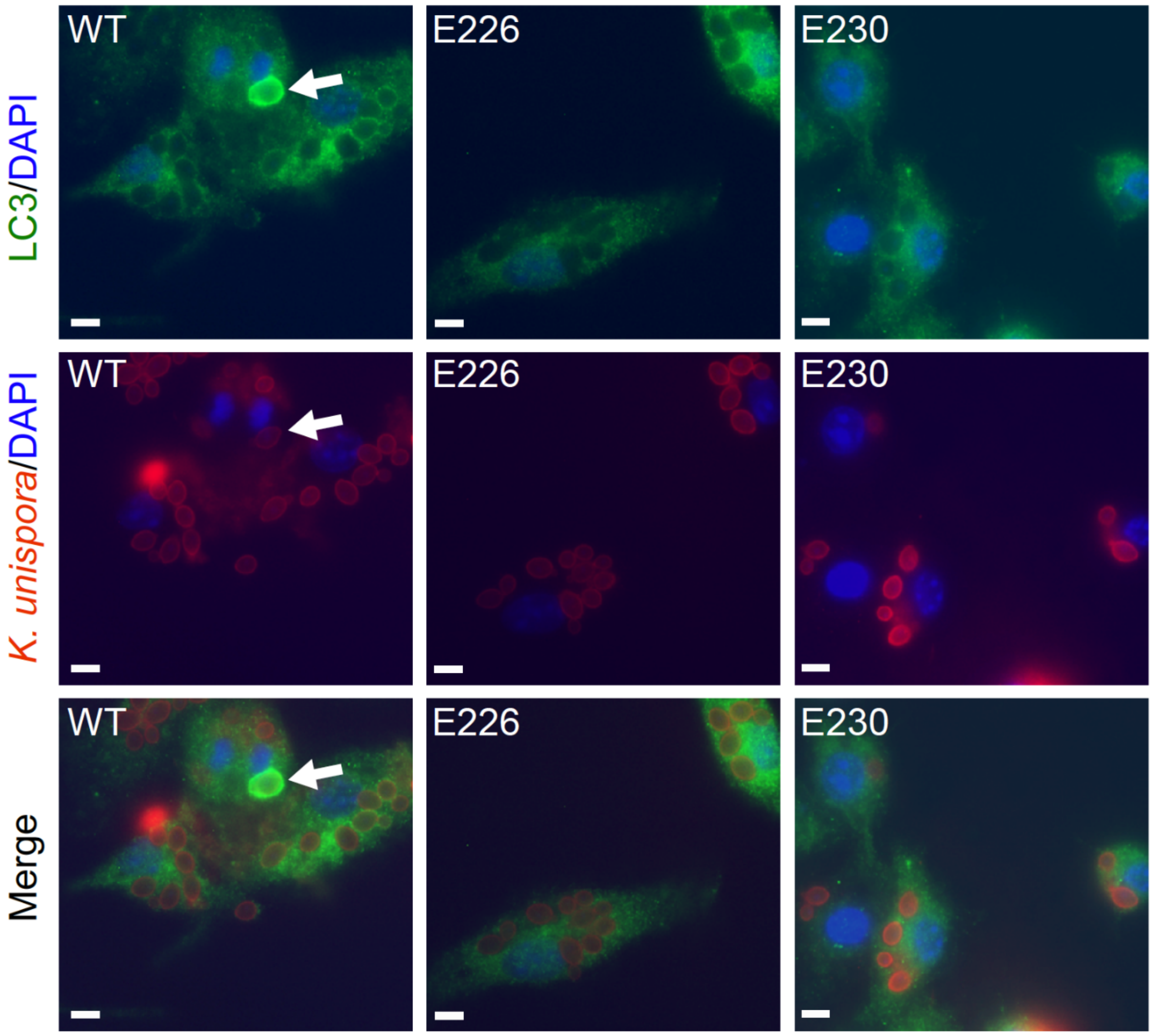

Supplementary Figure 2 *K. unispora* induces LAP in BMDCs.

WT, E226, and E230 BMDCs were incubated with Alexa555-labeled and PFA-fixed *K. unispora* at a MOI of 10 for 2 h. Cells were then fixed, permeabilized, and stained with anti-LC3 Ab and analyzed. White bar indicates 5 µm. Arrows show LC3 recruitment to the yeast. Data shown are representative photographs and reproducible in three independent experiments.

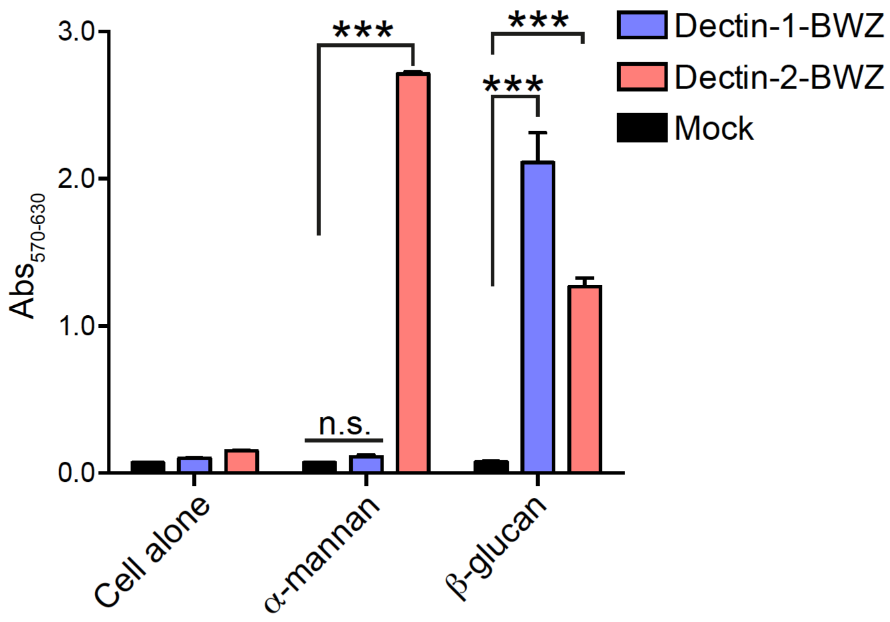

Supplementary Figure 3 α-mannan used in this study is specific to Dectin-2, but not to Dectin-1.

BWZ reporter cells expressing Dectin-1, Dectin-2, and mock transfectant were incubated with 50 μg/ml of α-mannan and β-glucan. After one day incubation, β-galactosidase activity in the reporter cells was monitored by a colorimetric assay. Data shown are the mean of triplicates ± SD from one representative experiment and reproducible in two independent experiments. Statistical analyses were performed by one-way ANOVA followed by Tukey's test.

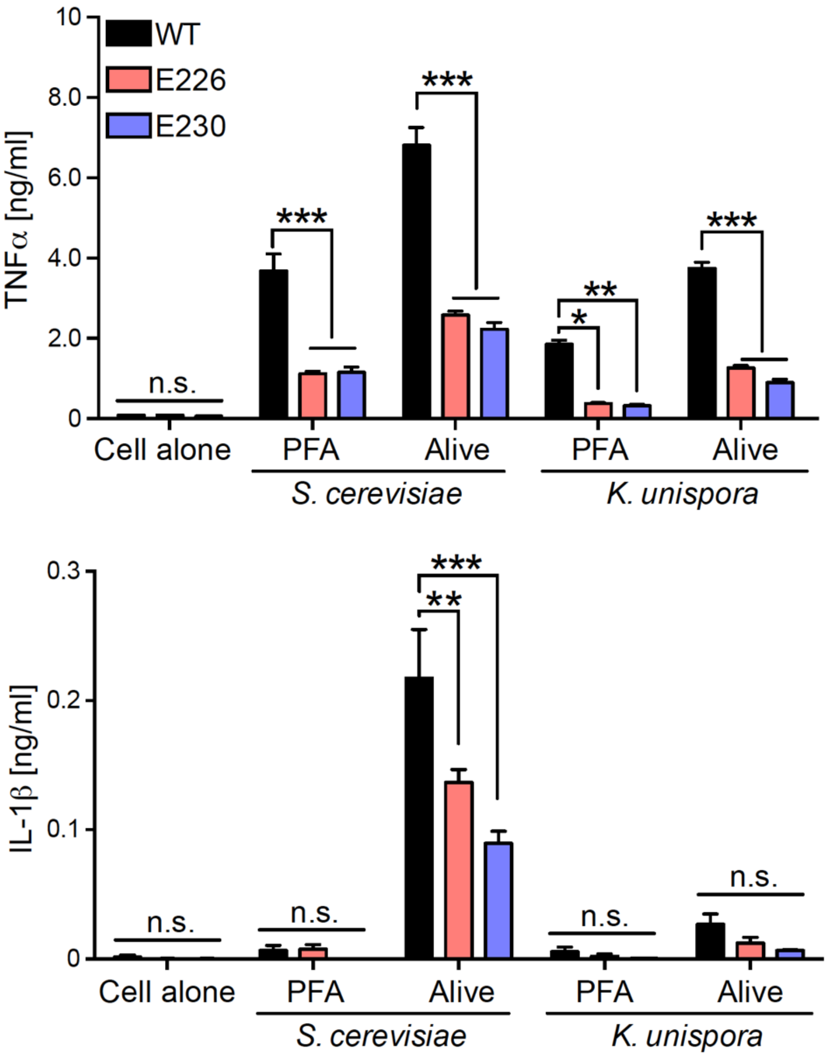

Supplementary Figure 4 PFA-fixed and living yeasts show the same trends in cytokine production.

WT, E226, and E230 BMDCs were stimulated with the indicated yeasts at a MOI of 5.0. TNFα and IL-1β in the culture supernatant was measured by ELISA. Data shown are the mean of triplicates ± SD from one representative experiment and reproducible in three independent experiments. Statistical analyses were performed by one-way ANOVA followed by Tukey's test.

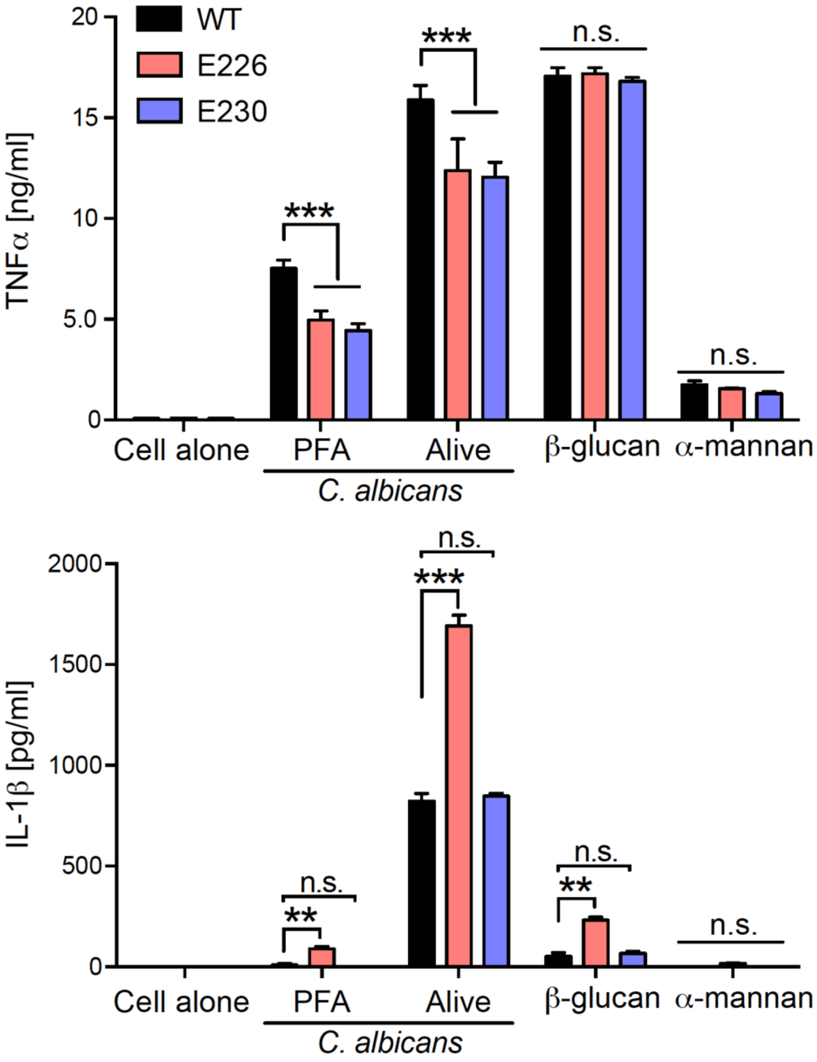

Supplementary Figure 5 BMDC response to *C. albicans* and fungal polysaccharides

WT, E226, and E230 BMDCs were stimulated with *C. albicans* at a MOI of 5.0, 1.0 mg/ml β-glucan, and 100 μg/ml α-mannan. TNFα and IL-1β in the culture supernatant was measured by ELISA. Data shown are the mean of triplicates ± SD from one representative experiment and reproducible in three independent experiments. Statistical analyses were performed by one-way ANOVA followed by Tukey's test.
